# Experimental Study on the Precise Orbit Determination of the BeiDou Navigation Satellite System

**DOI:** 10.3390/s130302911

**Published:** 2013-03-01

**Authors:** Lina He, Maorong Ge, Jiexian Wang, Jens Wickert, Harald Schuh

**Affiliations:** 1 Department of Surveying and Geo-informatics, Tongji University, Shanghai 200092, China; E-Mail: wangjiexian@mail.tongji.edu.cn; 2 Department of Geodesy and Remote Sensing, German Research Center for Geosciences, Potsdam 14473, Germany; E-Mails: maor@gfz-potsdam.de (M.G.); wickert@gfz-potsdam.de (J.W.); schuh@gfz-potsdam.de (H.S.)

**Keywords:** BeiDou, tracking network, precise orbit determination, ambiguity-fixing

## Abstract

The regional service of the Chinese BeiDou satellite navigation system is now in operation with a constellation including five Geostationary Earth Orbit satellites (GEO), five Inclined Geosynchronous Orbit (IGSO) satellites and four Medium Earth Orbit (MEO) satellites. Besides the standard positioning service with positioning accuracy of about 10 m, both precise relative positioning and precise point positioning are already demonstrated. As is well known, precise orbit and clock determination is essential in enhancing precise positioning services. To improve the satellite orbits of the BeiDou regional system, we concentrate on the impact of the tracking geometry and the involvement of MEOs, and on the effect of integer ambiguity resolution as well. About seven weeks of data collected at the BeiDou Experimental Test Service (BETS) network is employed in this experimental study. Several tracking scenarios are defined, various processing schemata are designed and carried out; and then, the estimates are compared and analyzed in detail. The results show that GEO orbits, especially the along-track component, can be significantly improved by extending the tracking network in China along longitude direction, whereas IGSOs gain more improvement if the tracking network extends in latitude. The involvement of MEOs and ambiguity-fixing also make the orbits better.

## 1. Introduction

China has been developing its own independent satellite navigation system for decades. Now the COMPASS system, also known as BeiDou, is emerging and gaining more and more attention in the worldwide GNSS communities. The system is designed as a global system, but with special concern for service in China and its surroundings [1]. Its development is scheduled into three phases: the demonstration system, the regional system, and the global system. Presumably, such a special schedule “from regional to global” will result in the utilization of Geostationary Earth Orbit (GEO) and Inclined Geosynchronous Earth Orbit (IGSO) satellites as a new feature of the BeiDou system. The demonstrational system was established as the BeiDou-1, with three GEO satellites providing positioning and short message communication services. The regional system comprises five GEOs, five IGSOs and four MEO satellites and provides positioning services for users in China and its surroundings. Afterwards, the whole constellation of the global system, which will consist of five GEOs, three IGSOs and 27 MEO satellites, is expected to be completed by the end of 2020 [1–3]. Up to now, the constellation of the regional system is completed and its operational service was officially announced by the BeiDou authorities at the end of 2012.

Due to the similar signal structure and analogous frequencies of BeiDou with respect to that of the American GPS and the European Galileo systems, BeiDou-capable multi-GNSS receivers were already developed by US and European manufacturers even before the Interface Control Document (ICD) was publicly disclosed. This enabled a number of investigations being carried out since the first experimental satellite M1 (C30) was launched in 2007. These research projects addressed the aspects of the signal decoding method [4], receiver hardware and software analysis [5], satellite visibility and dilution of position precision [6], precise relative positioning, and measurement quality analysis [3].

Precise orbit determination (POD) and precise clock determination (PCD) are essential functions of any global satellite navigation system. Their performance in terms of accuracy and time latency decides the capacity of the system services to some extent. Hence, POD and PCD of BeiDou are also hot topics for GNSS scientists as well. In general, a long orbit arc is needed for BeiDou POD using its own phase and range observations in order to obtain a stable solution because of the special constellation and the corresponding regional tracking network. The detailed dynamic and observation models are almost the same as for the GPS system except the phase center corrections of receivers and satellites are unknown, and satellite attitude control mechanics is not yet clear.

There are mainly two different strategies for POD data processing: (1) simultaneous observations from other systems or their derived products are strongly involved [2,7–9], and (2) only BeiDou data are employed [10,11]. In the former one, data from the other system, typically GPS, put very strong constraints on receiver clocks and tropospheric delays besides station coordinates, whereas the latter one is able to demonstrate the capacity of BeiDou as an independent navigation system. Using the first strategy Steigenberger *et al.* [9] presented POD results with an accuracy of a few meters for GEOs and 10 to 20 cm for IGSO satellites with data from an Asian-Pacific regional tracking network comprising six stations out of the Chinese territory. In their study, the impacts of both data arc length and parameterization of radiation pressure force model are also investigated. As most of the scientists pay more attention on the performance of BeiDou system alone as an independent system, POD and PCD are carried out using only BeiDou data from a regional tracking network consisting of about twelve stations [10,11]. The results confirm that orbit accuracy in 3D-RMS is better than 3 m for GEOs and 20 cm for IGSOs, and the accuracy of satellite clocks is 0.23 ns in STD and 0.56 ns in RMS. The products are validated by being applied to Precise Point Positioning (PPP) in both static and kinematic mode.

From the current achievements, there must be a large space for improvement on BeiDou POD, especially for the GEOs, due to the weak tracking geometry of the regional constellation. Montenbruck *et al.* [12] have also considered that substantial progress in the quality of BeiDou products can be expected in the future from a densified tracking network, the ambiguity fixing application and available parameters of the space segments. The BeiDou Experimental Test Service (BETS) network deployed by the GNSS research center at Wuhan University and including stations not only in China, but also worldwide, provides an opportunity for experimental studies on the above-mentioned issues. Hence, in this contribution we investigate the impact of network coverage on the POD products by comparing results from tracking networks over the Chinese territory, Asian-Pacific, Asian area and at a global scale. Furthermore, POD results with and without MEOs are compared to estimate the improvement of involving MEOs. Finally, integer ambiguity resolution, which brings significant improvement on orbits and positions with GPS data, is also carried out and its effect on POD products is assessed and discussed in detail.

After an introduction of the satellite constellation and the ground tracking network used in the experimental study, the POD strategy and processing procedure are described in Section 3 with the aspects of the observation model, satellite dynamical model, and parameter estimation. Section 4 illustrates the data processing scheme for the impact study. Afterwards the results and their comparison are discussed in Section 5.

## 2. Experimental Data Set

In this section we will illustrate the details of the data set used for this study. It includes the constellation, the tracking network, data availability and quality, so that the tracking geometry is clearly revealed.

### 2.1. Satellite Constellation

The designed constellation of the BeiDou regional system is composed of 14 satellites, including five GEOs, five IGSOs, and four MEO satellites. Up to November 2012, the constellation of the second development phase has been completed to provide service for areas in China and its surroundings. The five GEO satellites are positioned at 140°E (G1), 80°E (G3), 160°E (G4), 58.75°E (G5), and 110.5°E (G6), respectively [1], with an inclination of 0.7°–1.7°. The IGSO satellites have an inclination of about 55° and are located at various longitude bands from 90° to 125°. The MEO satellites fly in 21,528 km orbit plane with a period of 12 h 53 m. All the three types of satellites transmit triple-frequencies navigation signals, *i.e.*, 1,561.098 MHz, 1,207.140 MHz and 1,268.520 MHz for the B1, B2 and B3 bands, respectively. As the B3 signal can only be accessed by authorized users, it was not available for this study. The details of the 16 satellites in space are shown in Table 1.

Among the 16 satellites, G2 is drifting unstably and unusable, and M1 was for signal testing and validation only and is no longer used because of its clock problem [13]. During the period of the test data (Section 2.3) satellites M5, M6, and G6 were not yet launched. Therefore, in total eleven operational satellites were involved in this experiment.

### 2.2. Tracking Network

**Figure 1.**
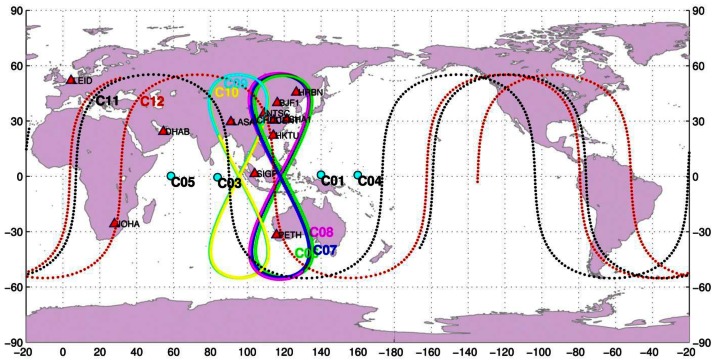
Ground tracks of the BeiDou satellites and distribution of the experimental tracking stations (for details see text).

The BETS network with BeiDou and GPS capacity has been deployed for scientific Positioning, Navigation and Time (PNT) service purposes. Since March 2011, 14 stations have already been established in China and its neighboring regions. Among these, 13 stations are employed in this contribution, eight of them located inside of China and five overseas. The stations in China are CENT in Wuhan, CHDU in Chengdu, HRBN in Harbin, HKTU at Hong Kong, NTSC and XIAN at Xi'an city, SHAO in Shanghai, and LASA in Tibet. The five overseas stations are SIGP (Singapore), PETH (Australia), DHAB (the United Arab Emirates), LEID (Netherlands), and JOHA (South Africa). The station distribution is shown in Figure 1. All the stations are equipped with the UR240 dual-frequency and GPS/BeiDou dual-system receivers and the UA240 antennas manufactured by the UNICORE Company in China [10]. As this is a newly developed receiver, some built-in attributes of the receiver antennas are unknown, for example, phase center offset (PCO) and phase center variation (PCV).

### 2.3. Data Set

More than seven weeks of tracking data from days 130 to 180 in 2012 were made available for this study by the GNSS Research Center at Wuhan University, with the permission of the BeiDou authorities. During this period, two satellites G2 and M1 were unavailable and three satellites G6, M5, and M6 were not yet launched. Therefore, there were eleven satellites in operation: four GEOs (C01, C03, C04, C05), five IGSOs (C06, C07, C08, C09, C10), and two MEOs (C11, C12). The ground tracks of the operational satellites are illustrated in Figure 1 together with the tracking stations for a better understanding of the observing geometry. For example, C01 and C04 are at the eastern edge and C03 and C05 on the western side of the tracking network, so the international stations on the western side improve the tracking geometry for C03 and C05 much more significantly than for C01 and C04. During the test time, maneuvers were detected on satellite C01 on days 149 and 179, C03 on 154, C04 on 144, C07 on 137, C08 on day 173, and C12 on 139.

**Figure 2.**
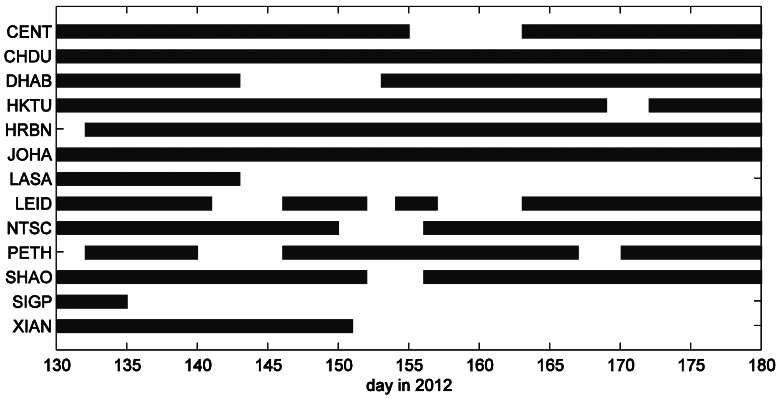
Daily data availability of the 13 selected tracking stations over the testing period.

Currently, the daily files are transferred from each station to the GNSS research center automatically. Details of data availability of each station during the selected test period are given in Figure 2. Because most of the stations were set up shortly before the data period and running in a test mode, long gaps exist due to hardware and software failures and communication problems as well. Several stations, for example, XIAN, SIGP, and LASA just have data at the beginning of the test period. On some days, there is half number of the stations without data, which should be considered carefully in the impact study of network geometry.

## 3. Precise Orbit Determination Strategy

The Position and Navigation Data Analyst (PANDA) Software [14,15] developed at the GNSS Research Center in Wuhan University is adapted for BeiDou data analysis for this study. The processing strategy including observation modeling, parameterization and satellite dynamic models, and processing procedure are discussed in this section.

### 3.1. Three-Day Solution

**Figure 3.**
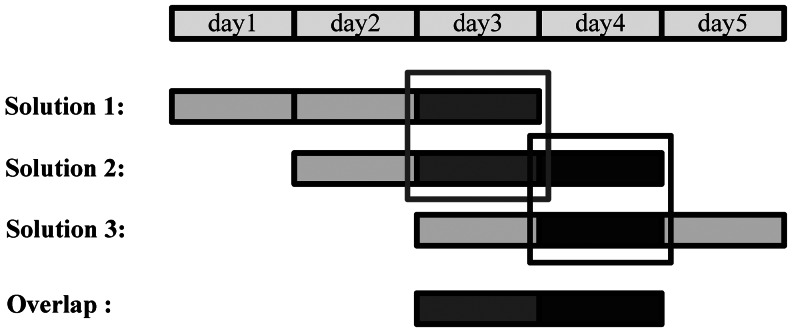
Three-day solution and orbit overlap comparison.

In order to obtain a stable solution, long data arc is needed for POD based on a regional tracking network because of the weak observing geometry. For the BeiDou regional system, GEO satellites have almost no movement with respect to the ground network and IGSOs are restricted within a certain longitude zone. Therefore, long arc estimation is even more important for the current regional BeiDou system. In this study we use three-day data in a batch estimation to obtain a three-day solution, instead of combining three daily solutions on the level of normal equations [9]. The orbit quality is assessed by the orbit consistency of two adjacent three-day solutions over the overlapping time: the orbit of the last day in one three-day solution is compared with that of the middle day in the next, as illustrated in Figure 3. Although the overlapping consistency, measured by the RMS of the orbit differences over the overlapping day, cannot fully represent the true orbit accuracy because two-thirds common data is involved in two adjacent solutions, from the validation using satellite laser range [9,11] it is still an useful orbit quality index for the related study.

### 3.2. Models

As the BeiDou system is very similar in signal structure and frequencies to GPS, the observation models and satellite force models for GPS can be utilized directly for BeiDou with very slight modifications. Therefore, similar observation models and dynamical models to the operational International GNSS Service (IGS) data processing at GFZ are selected for each three-day solution and they are listed in Tables 2 and 3, respectively.

### 3.3. Processing Procedure

**Figure 4.**
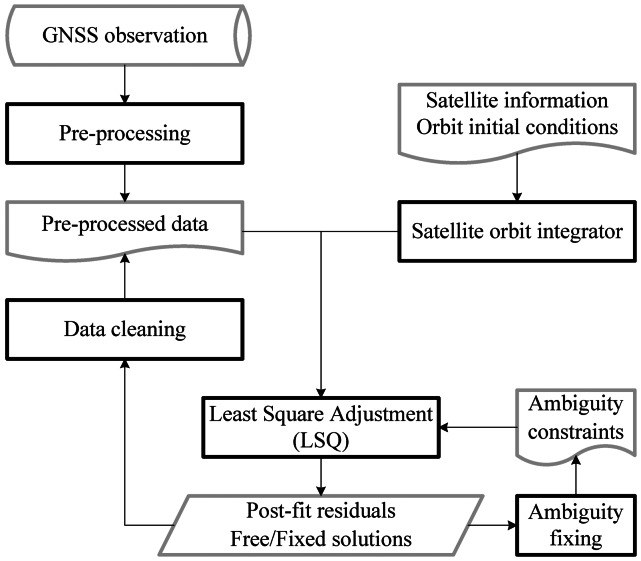
Procedure for precise orbit determination processing.

For each three-day solution, the processing procedure is illustrated in Figure 4. First of all, data pre-processing is carried out station by station to remove outliers and to flag cycle slips. Then, an initial orbit is generated by orbit integration. With the initial orbits and pre-processed observations least-squares adjustment is performed to estimate the parameters. Data editing based on post-fit residuals is undertaken to detect any possibly problematic observations. The last three steps must be run iteratively to obtain a free solution until the solution is converged with no more cycle slips and outliers are detected. Afterwards, ambiguity fixing can be carried out to obtain the fixed solution. After each adjustment, estimates including satellite orbits, station coordinates, and clocks of both stations and satellites should be updated for the next iteration or as final results.

It should be mentioned that for the newly launched MEOs M3 and M4, there were no broadcast navigation information. Their initial orbit conditions are estimated from BeiDou range observations with receiver clock and station coordinates derived from GPS.

## 4. Data Processing Scheme

There are a number of issues which have critical impact on POD of GNSS satellites, such as tracking geometry, force models, and estimating approaches. Thanks to the excellent activities of IGS, most of them are well-known. Here we concentrate on some of the issues which are special for the BeiDou regional system, and are achievable with the available data set. Aimed at possible improvements in BeiDou POD, we identified three topics for investigation: impact of the tracking network coverage, benefit of involving MEO satellites, and contribution of integer ambiguity-fixing. The corresponding data processing schemata are defined here and carried out for the selected data set and the results are discussed later on for possible further improvement.

### 4.1. Tracking Networks

The tracking network plays a very important role in POD. Thus IGS puts a large effort into optimizing its tracking network in terms of station density and distribution. In general, a tracking network with about 100 globally even distributed stations is used for POD of the GPS and/or GLONASS systems. For the BeiDou regional system, its constellation consists mainly of GEO and IGSO satellites whose movement is restricted over a dedicated region instead of around the Earth like MEOs. Obviously, these satellites can only be tracked by stations in a certain region and each station may contribute quite differently to different satellites. Therefore, the impact of tracking geometry is different from POD for GPS and further investigation is necessary for possible improvement. From Figure 1, the BETS network has five stations outside the Chinese territory. Among them SIGP and PETH extend the network towards the southern hemisphere and LEID, JOHA, and DHAB enlarge the network westward towards Europe and South Africa.

**Figure 5.**
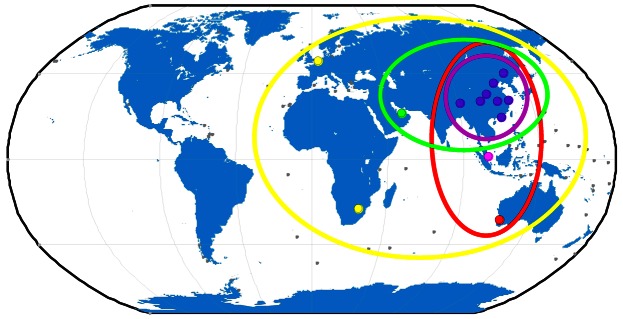
Tracking networks defined for the impact study of tracking geometry on satellite orbits. The Chinese regional network is indicated by a violet cycle, Asian-Pacific network in red, Asian network in green, and global network in yellow.

For the impact study of the tracking geometry, we selected four tracking networks displayed in Figure 5: the Chinese regional network (violet), Asian-Pacific network (red), Asian network (green), and the global network (yellow). The four networks will be processed with the same strategy and the orbits are compared to assess the impact of network geometry on satellite orbits.

### 4.2. Involvement of MEOs

According to the development schedule of BeiDou, GEOs and IGSOs are now the base of the current regional system and will still play a significant role for the region in the future global system. Nowadays, there are already four MEO satellites in operation and more and more will come into service. As MEOs can be tracked globally and their PODs can easily reach an accuracy of few cm from the IGS expertise, it is obviously an interesting question whether orbits of the GEO and IGSO satellites can be improved if MEOs are involved in POD processing.

In order to have a preliminary impression of such possible improvement, we process the global network with and without the two MEOs C11 and C12, respectively. The estimated GEO and IGSO orbits are assessed to show the effect of the involvement of MEOs. The result is presented in sub-Section 5.4.

### 4.3. Ambiguity-Fixing

As is well known, integer ambiguity resolution is critical in GPS data processing for obtaining the most accurate result. It improves orbit accuracy for GPS satellites significantly [18]. However, due to the very small movement of GEOs and IGSOs with respect to the tracking network, ambiguities could be biased differently, so that the integer property cannot be recovered by forming double-differenced ambiguity. Even if the ambiguities can be fixed to integer, its improvement on orbits is not definitely comparable to that of GPS. Because of the very small change of the tracking geometry, GEOs and IGSOs are usually tracked continuously or over a long time. Theoretically, there should be one ambiguity for each satellite-station pair in each solution. Then ambiguity estimates must be rather stable thanks to the long continuous data and the ambiguity-fixing may bring nearly no improvement. Anyway, we employ the fixing approach developed by [19] and adapted by [20] based on an ionosphere-free solution and the Melbourne-Wübbena combination. In this test, we try to fix ambiguities of different satellite types sequentially in order to confirm their fixing efficiencies and impact on satellite orbits. The details are in subsequent discussions in sub-Section 5.5.

## 5. Results

### 5.1. Measurement Quality

**Figure 6.**
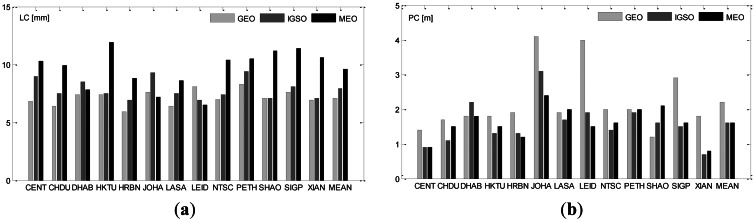
(**a**) The averaged RMS of LC observation residuals for each station-satellite pair. (**b**) The averaged RMS of PC observation residuals for each station-satellite pair.

The post-fit residual is a key indicator of accuracy or precision of observations and their modeling. RMS of the post-fit residuals of ionosphere-free range (PC) and phase (LC) observations are listed in Table 4 and illustrated in Figure 6. For each station, RMS of the three satellite types are shown for comparison.

From Table 4 or Figure 6, phase residuals of GEO, IGSO and MEO satellites are very similar and increase from 7 mm for GEOs to 10 mm for MEOs and the differences among stations are also very slight. However, the range observations of the stations located outside of China have a larger noise. JOHA has the largest RMS of about 4.1 m, 3.1 m and 2.4 m for GEO, IGSO and MEO satellites, respectively, and LEID has a very similar performance. The other three overseas stations DHAB, PETH and SIGP are rather close to China and show only a slightly larger RMS. Comparing with the results of Galileo [7], the phase accuracy of BeiDou is of a comparable quality, whereas range is slightly noisier.

**Figure 7.**
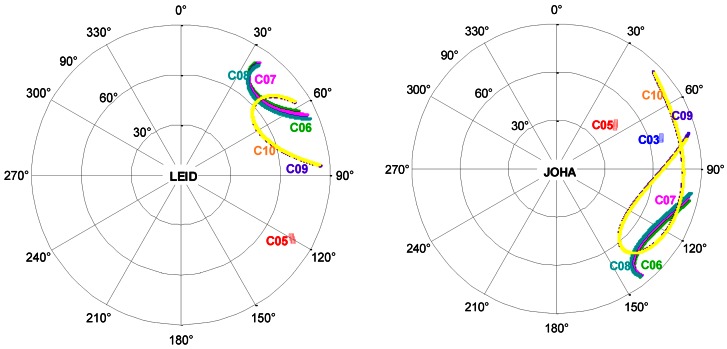

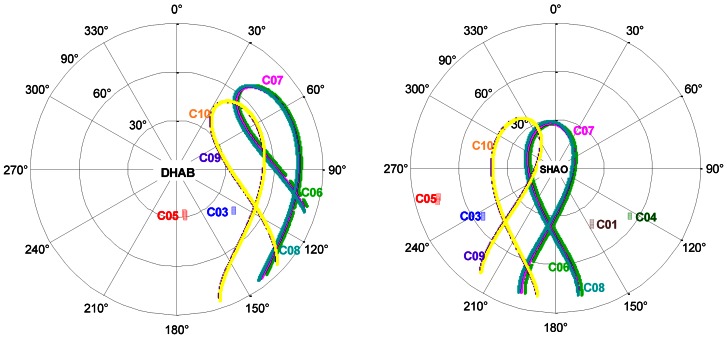
Sky plot of tracked satellites at four particular stations, LEID (51.9°, 44.0°), JOHA (−25.8°, 27.9°), DHAB (24.2°, 54.5°), and SHAO (121.5°, 30.9°) on day 171 in 2012. It is denoted by azimuth and zenith for four GEOs: C01, C03, C04, C05 and five IGSOs: C06, C07, C08, C09, C10. For some of the overseas stations, satellites can only be tracked on a rather low elevation and some of them are even not visible compared to station SHAO.

The distance-dependent range noise might be caused by the lower elevations of the GEO and IGSO satellites for the stations far away from China. As an example, Figure 7 provides a sky plot of the tracked GEO and IGSO satellites over one day at four particular stations for comparison: SHAO in China and three overseas stations: LEID, JOHA and DHAB. For the farthest station LEID the satellites come rarely above an elevation higher than 30 degrees and satellites C01, C03 and C04 are almost invisible. For station JOHA, the situation is slightly improved. At DHAB all satellites are tracked and even with a much higher elevation, but all satellites are on the east edge of the sky. This special satellite distribution and the low elevation might be the reason of the larger range RMS due to larger multi-path effects and inaccurate modeling of atmospheric delays.

### 5.2. Orbit Quality

In order to assess the quality of the estimated clocks and orbits, the differences over the overlapping time of two adjacent three-day solutions are utilized as usual. As shown on Figure 3, the orbit of the last day in a three-day solution is compared with that of the middle day of the next three-day solution. The RMS of the differences in along-track, cross-track and radial directions are taken as orbit quality indicator. The statistical results for orbits and clocks are listed in Table 5.

From the orbit RMS in Table 5, the along-track RMS is significantly larger than that of the other two directions, as expected. GEOs have the largest RMS in along-track direction of 114 cm compared to 24 cm and 45 cm for IGSOs and MEOs, respectively. RMS in cross-track and radial are very similar for the three types of satellites, *i.e.*, 10 cm and 6 cm for GEOs, 15 cm and 7 cm for IGSOs, and 13 cm and 12 cm for MEOs. In general, the orbit quality can still be further improved by optimizing the tracking geometry. For example, along-track RMS for GEOs can be reduced by extending the network. The larger RMS in across-track and radial for IGSOs and MEOs could be caused by inaccurate modeling of the satellite antenna phase center correction and the satellite attitude control.

### 5.3. Impact of Tracking Geometry

**Figure 8.**
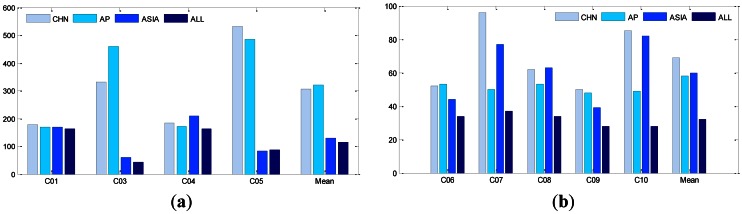
(**a**) 3D-RMS for GEOs of different networks. (**b**) 3D-RMS for IGSOs of different networks.

In order to investigate the impact of the tracking geometry, we defined four tracking networks: Chinese regional network (CHN), Asian-Pacific network (AP), Asian network (ASIA), and global network (ALL). The data are processed using an identical strategy. The resulting orbit overlaps RMS for each satellite over the seven weeks, are listed in Table 6, where the columns are sorted first by components then by networks. The 3D-RMS are also illustrated in Figure 8.

From the averaged 3D-RMS, in the AP network (adding PETH and SIGP to CHN network) the overlapping RMS for GEOs gets even slightly worse, but brings about 15% improvement for IGSOs. From Figure 1, it is obvious that PETH and SIGP enhance the tracking geometry to IGSOs significantly. Although the two stations also observe all the GEOs from the elevations to GEOs in Table 7 the observations can hardly strengthen the constraint in along-track, as these two stations locate in the same narrow longitude zone of the CHN network.

On the contrary, the ASIA network (adding DHAB to CHN network) extends the coverage of the CHN network to the west remarkably. Thus the 3D-RMS of GEOs drops from above 3.0 m to 1.3 m on average and IGSO orbits are also improved, but only slightly. Furthermore, from the RMS of each GEO satellite we notice that the 3D-RMS for C03 and C05 is reduced from several meters to decimeter-level, being very close to that of IGSOs, whereas very small changes for C01 and C04 are observed. If we examine the RMS in components, the improvement is taken place on the along-track direction. From the sky plot of DHAB in Figure 7 and the elevations in Table 7, C03 and C05 have a rather high elevation to DHAB while C01 and C04 are not visible because they are on the other (eastern) side and far away from DHAB.

For the ALL network with all the tracking stations, the RMS of IGSOs are reduced on average by about 50% compared with the other three networks, for example, 3D-RMS drops from about 60 cm to 32 cm. Compared with CHN and AP, ALL brings a dramatic improvement for C03 and C05 in along-track direction as ASIA does. There are about 10% further improvements in GEOs orbits compared to the ASIA network.

**Figure 9.**
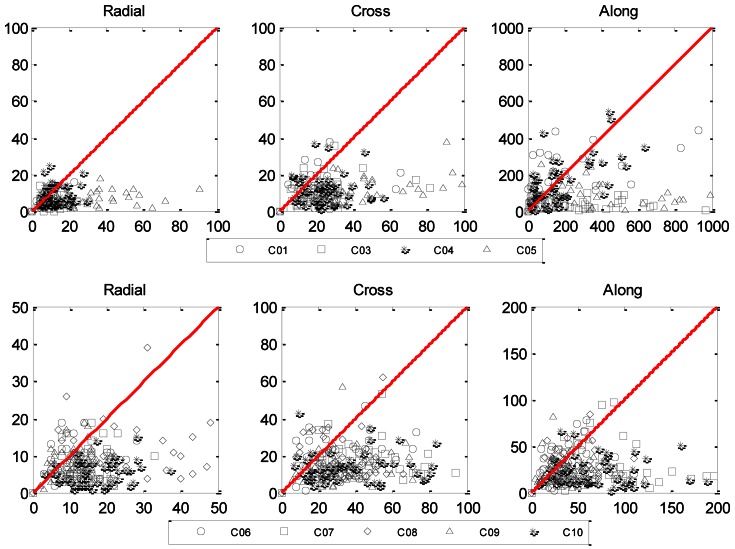
Relationship of the orbit RMS from the Chinese regional network (CHN) and the global networks (ALL). x-axis denotes the RMS of CHN, while y-axis represents orbit RMS of ALL. The red line with slope rate 1.0 divides each figure into two parts, of which the right down stands for the improvement.

Furthermore, Figure 9 shows the relationship of the orbit RMS of the ALL and CHN network with the upper panel for GEOs and bottom panel for IGSOs. On each sub-panel, *x*-axis is the orbit RMS of the CHN network and *y*-axis for that of the ALL network. Therefore, any point lays under the red diagram means an improvement on RMS by extending CHN to ALL. The closer a point lays to the *x*-axis, the larger the improvement rate is. A point very close to the origin means that the RMS in network CHN is rather small and it is not changed very much in the global network. A point far away from the origin and close to the *x*-axis means a significant improvement. From the plots in Figure 9, such improvement is obvious for both GEO and IGSO satellites.

### 5.4. Improvement of Including MEOs

**Figure 10.**
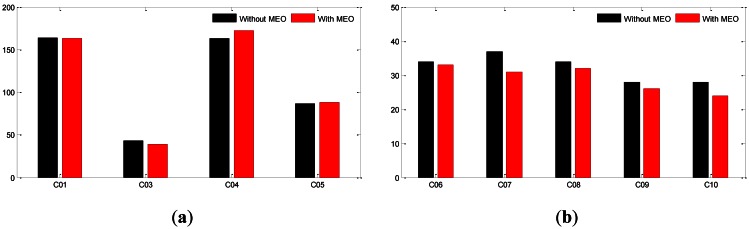
(**a**) 3D-RMS of GEO orbits estimated with MEOs and without MEOs. (**b**) 3D-RMS of IGSO orbits estimated with MEOs and without MEOs. Unit is cm.

As described in sub-Section 4.2, POD with and without MEOs are carried out using the global network (ALL). 3D RMS for GEOs and IGSOs of the two schemata are presented in Figure 10.

According to the 3D-RMS shown in Figure 10, the two MEOs bring almost no improvement on GEOs. On the contrary, a 10% improvement is found for IGSOs on average. Although the improvement is not as much as that of network geometry, it does further increase the orbit quality of the global network. Further investigations should be carried out if more simultaneously observed MEOs can be involved to provide stronger constraints on receiver clocks and tropospheric delay parameters.

### 5.5. Effect of Integer Ambiguity-Fixing

Considering the rather large orbit bias in the along-track direction of the GEO satellites and their poor tracking geometry, the double-differenced ambiguities of GEOs might not be fixed. Therefore, besides fixing ambiguities of all satellites, we also carried out ambiguity fixing of IGSOs and MEOs only to avoid any possible negative effect of GEOs.

On average there are approximately one to two ambiguities for each station-satellite pair for GEOs over the three-day session while IGSO or MEO has two to three times more. For both scenarios, the fixing percentages are almost the same of about 80% after two iterations.

For the scenario where all ambiguities are considered in the fixing procedure, satellite orbits become slightly worse than the free solutions in terms of the overlapping RMS. Unfortunately, we have not found any hint about the cause of this degradation and this topic thus remains under investigation. The major reason could be the poor tracking geometry that results in a large orbit bias in the along-track direction, up to several meters. Such orbit bias may contaminate ambiguities from this satellite to various stations but in a different way due to the different station locations. Consequently, the bias cannot be removed in the double-differenced ambiguities.

In the second scenario where only ambiguities of IGSO and MEO satellites are considered, ambiguity fixing shows a positive contribution from the overlapping RMS listed in Table 8 with that of the free solution for comparison. Compared RMS of the free and fixed solutions, 3D-RMS of IGSOs and MEOs are improved by 30% and 6%, respectively. The largest improvement occurs in the along-track direction.

## 6. Conclusions

With about seven weeks BeiDou data of the BETS network, BeiDou POD is carried out using the three-day solution strategy. The results are assessed by the orbit differences over the overlapped time span of the adjacent three-day solutions.

A number of processing scenarios are identified and data are processed to investigate the impact of tracking networks, by involving MEOs and by introducing integer ambiguity resolution for possible improvement on POD of the current BeiDou regional system.

From the post-fit observation residuals, BeiDou has similar phase accuracy as GPS and Galileo but a slightly larger range noise. In the tracking geometry investigation, extending the Chinese network to Australia brings rather small improvement on GEOs, whereas adding the United Arab Emirates station DHAB to the west of the Chinese network along-track RMS of C03 and C05 on the same side are reduced from several meters to decimeter level, but not for C01 and C04 on the eastern side as they are not observed from the newly added stations. Further improvement is also achieved if more western stations are included. From these results, we can conclude that deploying tracking stations on the eastern side, for example in New Zealand and/or in Hawaii will significantly reduce along-track RMS of C01 and C04.

Moreover, including the current two MEOs C11 and C12 brings further improvement on IGSO orbits by up to 10%, but no improvement on GEOs. Further tests should be carried out if more MEO satellites are available and involved.

Performing ambiguity-fixing to all satellites brings almost no improvement on the orbit quality. However, if only ambiguities of IGSOs and MEOs are considered, the along-track RMS is reduced. In general, ambiguity-fixing does not show a significant contribution as for GPS. This may be improved after a more stable and accurate free solution being achieved by a stronger tracking geometry.

## Figures and Tables

**Table 1.** Satellites of the current BeiDou constellation.

**Satellite**	**PRN**	**NORAD-ID**	**COSPAR-ID**	**Launch Date**	**Mean Longitude and Inclination**
G1	C01	36287	2010-001A	16/01/2010	140.0°E
G2	C02	34779	2009-017A	14/04/2009	Drift
G3	C03	36590	2010-024A	02/06/2010	80.0°E
G4	C04	37210	2010-057A	31/10/2010	160.0°E
G5	C05	38091	2012-008A	14/02/2012	58.75°E
G6	unknown	38953	2012-059A	25/10/2012	110.5°E

I1	C06	36828	2010-036A	31/07/2010	122°E (55°)
I2	C07	37256	2010-068A	17/12/2010	119°E (55°)
I3	C08	37384	2011-013A	09/04/2011	120°E (55°)
I4	C09	37763	2011-038A	26/07/2011	96.5°E (55°)
I5	C10	37948	2011-073A	01/12/2011	92.5°E (55°)

M1	C30	31115	2007-011A	13/04/2007	Discarded
M3	C11	38250	2012-018A	29/04/2012	55°
M4	C12	38251	2012-018B	29/04/2012	55°
M5	C13	38774	2012-050A	18/09/2012	55°
M6	C14	38775	2012-050B	18/09/2012	55°

**Table 2.** Summary of observation models and parameters applied in POD.

**Item**	**Models**
Observations	Undifferenced ionosphere-free code and phase combination of B1 and B2 with 60 seconds sampling
Elevation cutoff	7°
Weight for observations	Elevation dependent weight
Phase-windup effect	Applied [16]
Earth rotation parameter	Estimated with tight constraint
Tropospheric delay	Initial model + random-walk process
Ionospheric delay	Eliminated by ionosphere free combination
Satellite and receiver clock	White noise
Station displacement	Solid Earth tide, pole tide, ocean tide loading
	IERS Convention 2003 [17]
Satellite antenna PCO and PCV	Not corrected
Receiver antenna PCO and PCV	Not corrected

**Table 3.** Dynamic models involved for BeiDou orbit determination.

**Item**	**Models**
Geopotential	EGM96 model (12 × 12)
Tide	Solid Earth tide, pole tide, ocean tide
	IERS Conventions 2003
M-body gravity	Sun, Moon and all planets (JPL DE405)
Solar Radiation Pressure	Reduced BERN five parameters with no initial value
Relativistic Effect	Applied
Velocity breaks	Every other 12 hours
Attitude model	Assuming the same as GPS satellite of Block IIR

**Table 4.** Averaged RMS of the ionosphere-free phase (LC) and range (PC) observation residuals for each station-satellite pair.

	**LC (mm)**			**PC (m)**		
	
	GEOs	IGSOs	MEOs	GEOs	IGSOs	MEOs
**CENT**	6.8	9.0	10.3	1.4	0.9	0.9
**CHDU**	6.4	7.5	9.9	1.7	1.1	1.5
**DHAB**	7.4	8.5	7.8	1.8	2.2	1.8
**HKTU**	7.4	7.5	11.9	1.8	1.3	1.5
**HRBN**	5.9	6.9	8.8	1.9	1.3	1.2
**JOHA**	7.6	9.3	7.2	4.1	3.1	2.4
**LASA**	6.4	7.5	8.6	1.9	1.7	2.0
**LEID**	8.1	6.9	6.5	4.0	1.9	1.5
**NTSC**	7.0	7.4	10.4	2.0	1.4	1.6
**PETH**	8.3	9.4	10.5	2.0	1.9	2.0
**SHAO**	7.1	7.1	11.2	1.2	1.6	2.1
**SIGP**	7.6	8.1	11.4	2.9	1.5	1.6
**XIAN**	6.9	7.1	10.6	1.8	0.7	0.8
**MEAN**	7.1	7.9	9.6	2.2	1.6	1.6

**Table 5.** Averaged overlapping RMS of the estimated orbits and clocks for each individual satellites and the mean of each satellite type.

**Type**	**Satellites**	**Orbits (cm)**				**Clocks (ns)**	
	
		**Along**	**Cross**	**Radial**	**3D**	**STD**	**RMS**
GEO	C01	163	10	6	163	0.25	0.45
	C03	37	10	5	39	0.29	0.39
	C04	171	11	8	171	0.29	0.74
	C05	87	10	6	87	0.23	0.40

	Mean	114	10	6	115	0.27	0.49

IGSO	C06	27	17	7	33	0.31	0.39
	C07	26	16	7	31	0.18	0.25
	C08	25	17	10	32	0.32	0.39
	C09	21	14	7	26	0.29	0.35
	C10	20	13	5	24	0.43	0.49

	Mean	24	15	7	29	0.31	0.37

MEO	C11	48	13	12	51	0.43	0.48
	C12	42	12	11	45	0.39	0.44

	Mean	45	13	12	48	0.41	0.46

**Table 6.** Orbit RMS [cm] comparison for four networks: Chinese regional network (CHN), Asian-Pacific network (AP), Asian network (ASIA), and global network (ALL).

**Component**		**Along**				**Cross**				**Radial**				**3D**			

		ALL	ASIA	AP	CHN	ALL	ASIA	AP	CHN	ALL	ASIA	AP	CHN	ALL	ASIA	AP	CHN
GEO	C01	164	169	169	176	11	18	16	22	6	7	8	8	164	170	170	178
	C03	41	57	460	331	11	17	20	21	5	6	9	9	43	60	461	332
	C04	162	208	171	182	12	24	17	24	9	12	13	12	163	210	172	184
	C05	86	80	485	529	11	26	23	48	6	8	26	31	87	84	486	532

	Mean	113	128	321	304	11	21	19	29	7	8	14	15	114	130	322	306

IGSO	C06	28	36	41	39	17	23	32	33	8	11	11	12	34	44	53	52
	C07	31	69	40	83	18	31	29	45	7	14	10	15	37	77	50	96
	C08	27	50	43	48	18	28	26	32	11	26	18	24	34	63	53	62
	C09	23	30	39	36	14	22	26	33	8	10	10	11	28	39	48	50
	C10	24	75	39	73	14	28	27	40	6	17	11	17	28	82	49	85

	Mean	27	52	49	56	16	26	28	37	8	16	12	16	32	60	58	69

**Table 7.** GEOs elevation (in degrees) for tracking stations. Red cross for not visible.

	**C01**	**C03**	**C04**	**C05**
DHAB	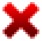	46	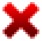	61
PETH	43	40	30	19
JOHA	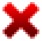	22	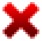	44
LEID	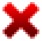	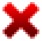	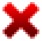	13
SIGP	48	66	26	38

**Table 8.** Orbit RMS for IGSO and MEO satellites of free and fixed solutions, unit is cm.

**Satellite**	**Solution**	**Along**	**Cross**	**Radial**	**3D**
IGSO	Free	24	15	7	29
	Fixed	14	13	7	20

MEO	Free	45	13	12	48
	Fixed	41	14	12	45
